# Potential Molecular Targets in the Treatment of Patients with CNS Tumors

**DOI:** 10.3390/cancers15153807

**Published:** 2023-07-27

**Authors:** Edward Pan

**Affiliations:** Daiichi-Sankyo, Inc., 211 Mt. Airy Road, Basking Ridge, NJ 07920, USA; epan@dsi.com

The challenges in identifying effective therapies for CNS tumors continue to be daunting. Potentially effective targeted therapies must be able to penetrate the blood–brain barrier to reach the tumor, and in sufficient concentrations to result in meaningful treatment responses. Moreover, molecular targets must be key drivers in the growth and progression of CNS tumors. Numerous potentially efficacious therapies have failed in randomized clinical trials due to other factors, including subclonal genetic intratumoral heterogeneity (particularly within malignant gliomas), epigenetic heterogeneity, and failure to target important factors involved in the tumor microenvironment. Developing effective targeted therapies requires a thorough fundamental understanding of the genetic and epigenetic factors driving tumor progression, the interactions between CNS tumor cells and the tumor microenvironment, and the key mechanisms of tumor treatment resistance.

In this Special Issue entitled “Updates on Molecular Targeted Therapies for CNS Tumors”, experts in the field of CNS tumors highlighted promising molecular targets in the development of treatments for patients with CNS tumors. The scope of this Special Issue includes multiple types of CNS tumors, translational and clinical studies, various treatment approaches (e.g., systemic therapies, radiotherapy, immunotherapies, etc.), as well as high-level reviews. 

Brain metastases (BM) are the most common CNS tumors, with an estimated incidence of up to 40% in patients with metastatic cancer [1,2]. The most common solid tumor BM arises from lung cancer [3]. Tatineni et al. evaluated the efficacy of first versus third-generation EGFR TKIs in EGFR-mutated NSCLC BM in both first line and later line treatments [4]. Although no survival benefits between the first- and third-generation EGFR TKIs were found, larger prospective studies to confirm these findings are warranted. In another study, Tatineni et al. evaluated the combination of EGFR-Directed TKIs with radiotherapy in patients with NSCLC BM [5]. They found that these patients treated with EGFR TKIs plus stereotactic radiosurgery (SRS) had higher OS compared to those BM patients treated with EGFR TKIs plus whole brain radiation therapy (WBRT), suggesting that larger phase II/III clinical trials are warranted to investigate the synergy of EGFR TKIs with SRS in EGFR-mutated NSCLC BM. Sharma et al. reviewed other potential molecular targets (e.g., ALK, ROS-1, HER-2, etc.) in a tumor-agnostic fashion for BM harboring these specific mutations [6]. This article illustrates the need for continued evaluation of tumor tissue for their molecular profiles in addition to histologic diagnosis to improve our understanding of the molecular nature of BM. 

Glioblastoma (GBM) is the most frequent primary malignant brain tumor in adults, with an incidence of 3–4 cases per 100,000 population, and often with poor prognoses (1 year survival rate of approximately 41%) [7]. Thus, there needs to be significant advances in our understanding of the molecular landscape of GBMs in order to make more meaningful clinical advances in GBM treatment. Georgescu described a multi-platform classification of an adult GBM cohort [8]. The study identified seven non-redundant IDH-wild type GBM molecular subgroups corresponding to the upstream RTK and RAS-RAF segment of the ERK/MAPK signal transduction pathway. Thus, this pathway may be utilized for potential targeted therapy approaches to GBMs. Singh et al. reviewed the role of T cell chemotaxis and infiltration in GBM [9]. This review discusses this process and the potential immunotherapeutic approaches to enhance T cell trafficking in GBM tumor cells, such as combinations of small-molecule inhibitors of the AKT1 and AKT2 isoforms with novel bispecific constructs with immune stimulatory cytokines. Bova et al. reviewed the role of adenosine and its interaction with its subtype receptions, as well as the potential efficacy of adenosine receptor antagonists (e.g., selective A2A receptor antagonists) to enhance immunotherapy effects in GBMs [10]. Moretti et al. analyzed the potential of targeting the metabolic status and tumor microenvironment in GBMs, specifically TLR4, in GBM cell lines. Metformin in combination with temozolomide (TMZ) demonstrated a response to a particular GBM cell line subtype with an activated TLR4 pathway, while another GBM cell line subtype (mitochondrial) with concomitant *CXCL8*/IL8 upregulation was more likely to respond to metformin combined with an antioxidant inhibitor (e.g., anti-SOD1) [11]. Thus, further exploration of the metabolic and antioxidant status of GBMs may yield another viable targeting strategy for GBMs. Another potential strategy is to decrease the resistance of GBMs to radiotherapy, which is currently the most effective treatment modality for GBM patients [12]. Tallman et al. evaluated the potential to increase sensitivity of GBM cells in mitosis to localized radiotherapy. A small molecular inhibitor of KIF11 (ispinesib) combined with radiotherapy demonstrated increased apoptosis in vivo compared to control plus radiotherapy [13]. Thus, the potential efficacy of ispinesib should be explored in GBM clinical trials. Nagane et al. completed a phase II trial that explored the effect of bevacizumab beyond progression in newly diagnosed GBM patients and evaluated predictors of response to bevacizumab. Although the primary endpoint was not met (2-year survival rate of 27%), RNA expression profiling identified Cluster 2 (enriched with genes involving microglia or macrophage activation) study patients as having longer OS and PFS independent of *MGMT* methylation status [14]. Thus, consideration may be given to complete a clinical trial evaluating bevacizumab in GBMs with the Cluster 2 subtype to determine if these specific GBM patients may derive increased benefit from antiangiogenic therapies.

Although low-grade gliomas (LGG) are less common (30% of all CNS tumors) and have better prognoses compared to GBMs, they eventually progress to high-grade gliomas and are ultimately fatal, with 5-year survival rates ranging from 30 to 80% [15]. Thus, there is an unmet need to develop novel therapeutics for patients with LGG. Dasgupta et al. reviewed the preclinical in vitro and in vivo models of LGG [16]. The review highlights the mechanistic challenges in generating accurate LGG models and summarizes potential strategies to overcome these challenges. Ozair et al. reviewed the role of epigenetics (specifically DNA methylation and histone modification) in LGG. This review summarizes the potential diagnostic and therapeutic targets for LGG (e.g., PARP, IDH, TERT, etc.) as well as the current clinical trial landscape for this patient population [17]. 

IDH-mutated gliomas have a distinct tumor biology compared to IDH-wildtype gliomas at both the genetic and epigenetic levels [18], with IDH-mutated gliomas having significantly more favorable outcomes compared to IDH-wildtype gliomas [19]. Yu et al. evaluated the association between tumor mutational burden (TMB), expressed neoantigens, and the tumor immune microenvironment in both IDH-mutant and IDH-wildtype gliomas to determine whether TMB may be a potential biomarker in diffuse gliomas [20]. The analysis of glioma samples determined that TMB was inversely correlated with immune score in IDH-wildtype gliomas with no correlation in IDH-mutant gliomas, suggesting further analyses of germline variants in a larger glioma cohort are warranted.

Ependymomas, although histologically classified as gliomas, behave differently from the typical gliomas. They originate from the lining of cerebral ventricles, occur more frequently in children than adults, are usually more chemotherapy-resistant, and have a different grading system than those of gliomas [21]. Larrew et al. discussed the molecular classifications of ependymomas and described potential therapeutic targets for patients with ependymomas based on their molecular classification (e.g., anti-YAP, FGFR3, anti-RELA, etc.) [22]. 

Primary CNS Lymphoma (PCNSL) is a rare variant of extra-nodal non-Hodgkin lymphoma affecting the CNS and/or vitreoretinal space without systemic involvement. It affects approximately 1600 people in the U.S. per year with a median age of diagnosis at 67 years [23]. Despite PCNSL typically being sensitive to chemotherapy and radiotherapy, relapse rates are high, especially for those who are not candidates for high-dose chemotherapy followed by autologous stem cell transplant, approximately 15% of patients have refractory disease, and median survival after first relapse is only 4.5 months [24]. Schaff and Grommes reviewed potential novel therapeutics for PCNSL, including targeting the BCR/TLR pathway, PI3K/mTOR pathway, and immunomodulatory drugs [25].

This Special Issue also includes studies involving pediatric CNS tumors. The most common malignant childhood brain tumor is medulloblastoma. Survival outcomes significantly depend on the molecular genetics and epigenetics of the medulloblastoma subtype [26]. Pham et al. discussed their metabolic studies of *MYC*-amplified medulloblastomas both in vitro and in vivo. They demonstrated that these specific medulloblastomas had upregulation of the TCA cycle and were dependent on several potentially targetable metabolic pathways, including tricarboxylic acid, amino acid, and hexosamine [27]. Another CNS tumor afflicting primarily children are the diffuse midline gliomas (DMG), which include diffuse intrinsic pontine gliomas (DIPG). They typically arise in the brainstem, thalamus, spinal cord, and cerebellum, which do not often allow for safe aggressive resections. They generally have dismal prognoses, with 5-year survival rates of less than 1% due to their high resistance to chemotherapy and radiotherapy, as well as their origins deep in the CNS structures [28]. Hayden et al. reviewed the underlying molecular landscape of DMG and discuss potential treatment targets, including HDAC, BET, and cell cycle inhibitors [29]. 

Finally, this Special Issue also includes reviews of rare neurologic diseases involving cancer, specifically neurofibromatoses and neoplastic meningitis. The neurofibromatoses, encompassing NF1, NF2, and schwannomatosis, are genetic tumor syndromes which cause affected patients to develop characteristic nerve-associated tumors both in the CNS and PNS (peripheral nervous system). Sanchez et al. review the clinical and molecular landscape of neurofibromatoses and discuss the recent treatment advances, particularly MEK inhibition with selumetinib and other potential therapeutic targets [30]. Neoplastic meningitis (NM) involves the spread of a primary tumor to the leptomeninges, dura, and subarachnoid space. The incidence of NM ranges from 5–8% (solid tumors) to 15% (hematologic malignancies), and typically has dismal prognoses with an overall survival of 2–4 months from diagnosis with treatment [31]. Khosla et al. reviewed the pathophysiology and current clinical trial landscape and highlighted potential targeted and immunotherapy strategies for the treatment of NM [32]. 

The goals of this Special Issue are to illustrate the various CNS tumor types and syndromes in both the adult and pediatric population and to highlight the shift in treatment strategies from traditional chemoradiotherapy approaches to target the key molecular drivers in these tumors. Increasing our understanding of the complex interactions within tumor cells as well as those of these cells with their tumor microenvironment will be crucial to the development of effective treatments for CNS tumors.
